# Exploiting the Multifaceted Effects of Cannabinoids on Mood to Boost Their Therapeutic Use Against Anxiety and Depression

**DOI:** 10.3389/fnmol.2018.00424

**Published:** 2018-11-20

**Authors:** Mario Stampanoni Bassi, Luana Gilio, Pierpaolo Maffei, Ettore Dolcetti, Antonio Bruno, Fabio Buttari, Diego Centonze, Ennio Iezzi

**Affiliations:** ^1^Unit of Neurology and Neurorehabilitation, IRCCS Neuromed, Pozzilli, Italy; ^2^Laboratory of Synaptic Immunopathology, Department of Systems Medicine, Tor Vergata University, Rome, Italy

**Keywords:** endocannabinoids, reward, stress, depression, anxiety, multiple sclerosis, EAE, inflammation

## Abstract

The endocannabinoid system (ECS) has been recently recognized as a prominent promoter of the emotional homeostasis, mediating the effects of different environmental signals including rewarding and stressing stimuli. The ECS modulates the rewarding effects of environmental stimuli, influencing synaptic transmission in the dopaminergic projections to the limbic system, and mediates the neurophysiological and behavioral consequences of stress. Notably, the individual psychosocial context is another key element modulating the activity of the ECS. Finally, inflammation represents an additional factor that could alter the cannabinoid signaling in the CNS inducing a “sickness behavior,” characterized by anxiety, anhedonia, and depressive symptoms. The complex influences of the ECS on both the environmental and internal stimuli processing, make the cannabinoid-based drugs an appealing option to treat different psychiatric conditions. Although ample experimental evidence shows beneficial effects of ECS modulation on mood, scarce clinical indication limits the use of cannabis-based treatments. To better define the possible clinical indications of cannabinoid-based drugs in psychiatry, a number of issues should be better addressed, including genetic variability and psychosocial factors possibly affecting the individual response. In particular, better knowledge of the multifaceted effects of cannabinoids could help to understand how to boost their therapeutic use in anxiety and depression treatment.

## Endocannabinoid system

The endocannabinoid system (ECS) consists of endocannabinoids (eCBs), cannabinoid receptors (CBRs), biosynthesizing, and degrading enzymes. The main eCBs, anandamide (AEA) and 2-arachidonoyl-glycerol (2-AG), are degraded by distinct enzymes, the fatty-acid amide hydrolase (FAAH) and monoacylglycerol lipase (MAGL), respectively. eCBs interact with a number of different molecular targets (Howlett et al., 2002) as the two CBRs (CB1R and CB2R) and with other G protein-coupled receptors (GPCRs) including GPR55 and GPR119, GPR18 which are expressed in different tissues, including the immune cells, and mediate a wide range of physiological functions (Chiang et al., 2015; Morales and Reggio, 2017). Furthermore, eCBs bind to other receptor subtypes, as the transient receptor potential vanilloid type 1 (TRPV1) cation channel (Tóth et al., 2009), the peroxisome proliferator-activated receptor, and glycine receptors (Zhang and Xiong, 2009). Finally, it has been demonstrated that CBRs can form heterodimers with other receptors including serotonin (5-HT), opioid and dopamine (DA) receptors (Farran, 2017).

Numerous physiological functions, as mood, cognition, feeding behavior and pain perception, are modulated by the ECS (Di Marzo et al., 1998; Di Marzo, 2009; Castillo et al., 2012; Pacher and Kunos, 2013). CB1Rs represent the most numerous GPCRs in the adult brain, particularly expressed in regions involved in reward, addiction and cognitive functions, including the amygdala, cingulate cortex, prefrontal cortex, ventral pallidum, caudate, nucleus accumbens, ventral tegmental area, and lateral hypothalamus (Glass et al., 1997; Wang et al., 2003). Synaptic transmission is controlled by the eCBs through a physiological feedback mechanism acting to avoid excessive synaptic excitation or inhibition (Lovinger, 2008). In particular, the eCBs act as retrograde messengers (Wilson and Nicoll, 2002) suppressing neurotransmitter release either at ɤ-aminobutyric acid (GABA)ergic or glutamatergic synapses (Alger, 2002; Heinbockel et al., 2005). Furthermore, as CB1Rs are mainly located on the presynaptic terminals, eCBs may directly modulate other neurotransmitter pathways as opioid peptides, acetylcholine and 5-HT (Heifets and Castillo, 2009; Kano et al., 2009).

**Figure 1:**
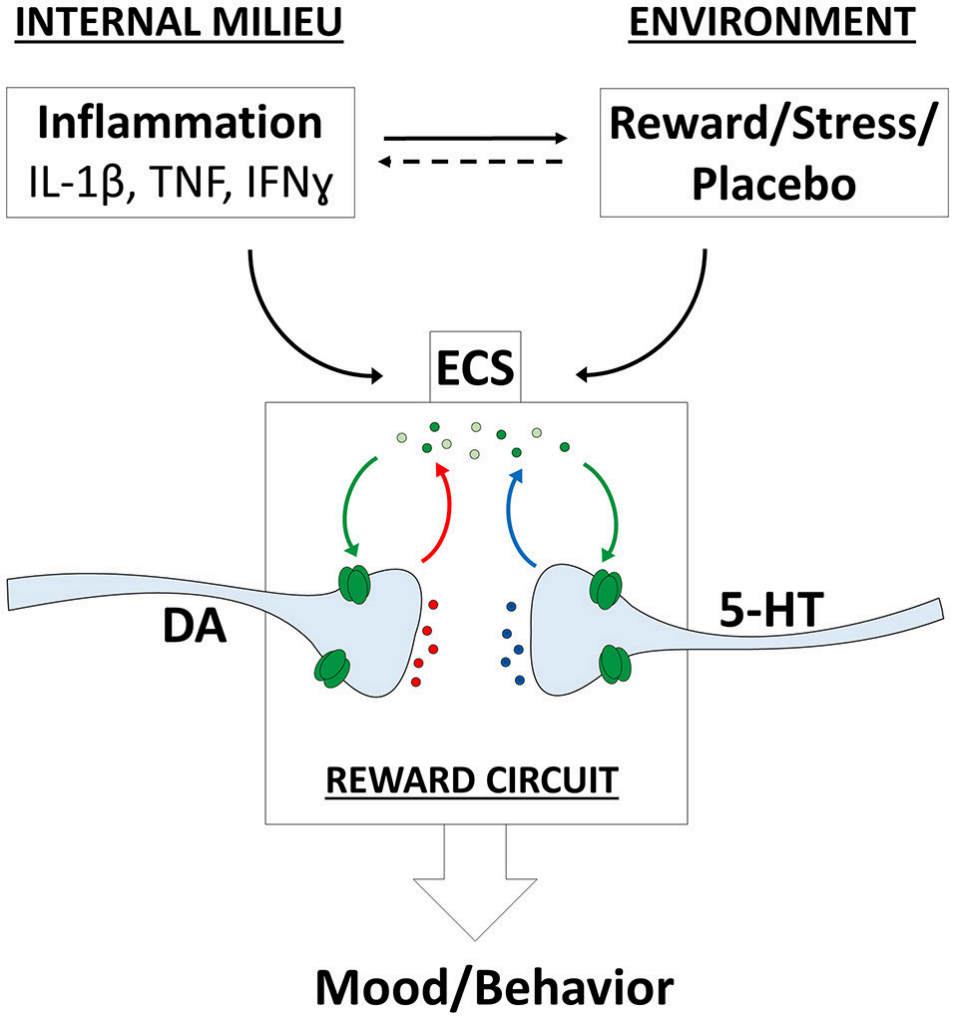
Endocannabinoid system (ECS) influences on mood and behavior. Complex relationships between internal and environmental factors converge on the ECS and modulate mood and behavior. The eCBs modulate different neurotransmitter systems including dopaminergic (DA) and serotoninergic (5-HT) projections onto the reward circuit. IFN-ɤ, interferon ɤ; IL-1β, interleukin-1β; TNF, tumor necrosis factor.

The ECS have a prominent role in maintaining emotional homeostasis, mediating the effects of different environmental signals, including rewarding and stressing stimuli (Parsons and Hurd, 2015). Moreover, recent evidence showed that also the immune system interacts with the ECS. In particular, different inflammatory mediators alter CB signaling in the CNS (Figure 1).

## ECS control of emotions and behavior

The ability to integrate external and internal stimuli to select appropriate behaviors is crucial for individuals. The ECS regulates a number of physiological functions and mediates the crosstalk between different neurotransmitter systems, therefore representing a key player in the control of behavioral responses (Lutz et al., 2015). Indeed, a number of emotions and behaviors, such as fear, anxiety, depression, stress-coping and reward-driven behaviors are critically modulated by the ECS.

In particular, it has been demonstrated that CB1Rs are expressed in brain areas critically involved in the control of mood and behavior, including limbic and frontal regions, and regulate serotonin transmission (Herkenham et al., 1991; Nakazi et al., 2000). Preclinical and human studies evidenced that activity of the ECS regulates anxiety and depression and modulates behavioral responses to stress and reward (Table 1).

**Table 1:** Experimental and human studies showing the role of the ECS in the regulation of mood and in mediating the responses to reward, stress and inflammation.

**Author**	**Experimental protocol/model**	**Main findings**
Navarro et al., 1997	Defensive withdrawal test, elevated plus-maze	CB1R antagonist administration induced anxiety-like responses in both protocols, without influencing the pattern of horizontal locomotor activity and the total activity in the elevated plus-maze. The secretion of stress related hormones was not influenced.
Haller et al., 2004	Elevated plus-maze Wild type (WT) and CB1R-KO mice	CB1R-KO showed higher anxiety-like behavior than the WT. Administration of the CB1R antagonist AM251 increased anxiety-like behavior in WT but had no effect in the CB1R-KO. The CB agonist WIN-55,212-2 reduced anxiety-like behavior. The effect of WIN-55,212-2 was abolished by CB1-antagonist only in WT.
Hill and Gorzalka, 2005b	Forced swim test (FST)	Desipramine reduced immobility duration in the FST. Immobility was significantly reduced by the administration of the eCB uptake inhibitor AM404, the CB1R agonist HU-210, and Oleamide. The effects of AM404, HU-210 and Oleamide were blocked by pretreatment with the CB1R antagonist AM251.
Hill et al., 2006	Desipramine, FST	Three-weeks administration of desipramine increased CB1R density in the hippocampus and hypothalamus. Chronic desipramine treatment reduced the secretion of corticosterone and the induction of the immediate early gene c-fos in the hypothalamus following a 5 min exposure to swim stress. Acute treatment with the CB1R antagonist AM251 before swim stress, blocked the effects of desipramine.
Domschke et al., 2008	CNR1 SNP, Major depression	CNR1 rs1049353 G allele was associated with increased pharmacoresistance to antidepressants. G allele was also associated with reduced bilateral activity in the amygdala, putamen and pallidum, and reduced activity in the left caudate and left thalamus in response to emotional faces.
Centonze et al., 2007	Cocaine consumption	Seven days of cocaine consumption induced sensitization of striatal GABA synapses to CB1R stimulation by HU-210. The CB1R-induced modulation of glutamate transmission was unaltered by cocaine.
De Chiara et al., 2010	Running wheel, Sucrose consumption	Running wheel and sucrose consumption induced sensitization of striatal GABA synapses to CB1R stimulation. Both wheel running or sucrose consumption prevented the effects of stress on striatal GABA synapses.
Chen et al., 2008	CNR1 SNP, Nicotine dependance	Variants and haplotypes in the CNR1 gene may play important roles in developing nicotine dependence. These associations seem to be sex specific.
Zuo et al., 2007	CNR1 SNP, Substance dependence	possession of both the G allele of the) 17937 T > G polymorphism (rs6454674) and the T/T genotype of the 4893 T > C (rs806368) has been recently associated with a higher risk of AD possession of both the G allele of the) 17937 T > G polymorphism (rs6454674) and the T/T genotype of the 4893 T > C (rs806368) has been recently associated with a higher risk of AD possession of both the G allele of the) 17937 T > G polymorphism (rs6454674) and the T/T genotype of the 4893 T > C (rs806368) has been recently associated with a higher risk of AD rs6454674 and rs806368 have been significantly associated with a higher risk of drug dependence and alcohol dependence.
Hohmann et al., 2005	Stress- induced analgesia (SIA)	Blockade of CB1Rs in the periaqueductal gray (PG) prevents non-opioid SIA. Stress increases 2AG and AEA levels in the PG. Both MAGL and FAAH inhibitors enhanced SIA.
Rademacher and Hillard, 2007	Restraint stress	Mice exposed to restraint showed reduced preference for sucrose over water. The effects of restraint stress were reduced by the CB agonist CP55940 or the FAAH inhibitor URB59, and enhanced by the CB1R antagonist rimonabant.
Rossi et al., 2008	Social defeat stress (SDS)	Stress exposure reduced the sensitivity of GABA synapses in the striatum to CB1R stimulation. This alteration was prevented by pharmacological blockade of glucocorticoid receptors and was reproduced by corticosterone injection. Running wheel, sucrose administration, or a single injection of cocaine promoted the recovery of synaptic defects.
Lu et al., 2008	CNR1 SNP, Attention deficit hyperactivity disorder (ADHD) and post-traumatic stress disorder (PTSD)	Significant associations were identified between CNR1 SNP haplotypes and both ADHD and PTSD.
Agrawal et al., 2012	CNR1 SNP, Anhedonia	History of childhood physical abuse was significantly associated with anhedonia only in rs1049353 GG patients.
Rossi et al., 2012	IL-1β injection, SDS	Both exposure to SDS and IL-1β injection induced anxiety and abolished the sensitivity of CB1R controlling GABA synapses. Injection of IL-1ra reverted both effects. The effects of IL-1β required intact function of the TRPV1 channel.
Gentile et al., 2016	EAE mice, CB1R-KO mice	EAE-induced anxiety was associated with reduced the sensitivity of GABA synapses in the striatum to CB1R stimulation. These alterations were exacerbated in CB1R-KO mice. IL-1ra administration reduced anxiety in EAE mice, and restored both DA release and the sensitivity of striatal GABA synapses to CB1R stimulation.
Mandolesi et al., 2017	IFN-ɤinjection, EAE mice	IFN-ɤinjection induced anxiety and depression, associated with reduced sensitivity of striatal GABA synapses to CB1R stimulation. EAE was associated with increased striatal IFN-ɤexpression and altered CB1R transmission. These alterations were rescued by blocking IFN-ɤsignaling.

*ADHD, attention deficit hyperactivity disorder; CB, cannabinoid; CB1R, cannabinoid receptor 1; CB1R-KO, cannabinoid receptor 1-knock out; DA, dopamine; EAE, experimental autoimmune encephalomyelitis; ECS, endocannabinoid system; FAAH, fatty acid amide hydrolase; FST, forced swim test; GABA, ɤ-aminobutyric acid; IFN-ɤ, interferon ɤ; IL-1ra, interleukin-1 receptor antagonist; IL-1β, interleukin-1β; PTSD, post-traumatic stress disorder; SDS, social defeat stress; SIA, stress-induced analgesia; TNF, tumor necrosis factor; WT, wild type*.

### The ECS regulates depression and anxiety

Preclinical studies evidenced that reduced ECS activity induces depressive-like behaviors in animals in different experimental paradigms usually employed to test the antidepressant effects of drugs (Martin et al., 2002; Hill and Gorzalka, 2005a). In particular, CB1R-knock out (CB1R-KO) mice showed increased immobility in the forced-swim test (Uriguen et al., 2003), higher sensitivity to depressive-like responses in the chronic unpredictable mild stress paradigm and increased anxiety-like behavior in the light/dark test (Martin et al., 2002). Moreover, administration of CB1R antagonists increased anxiety-like behavior in the defensive withdrawal test (Navarro et al., 1997) and in the elevated-plus maze test (Navarro et al., 1997; Haller et al., 2004; Patel and Hillard, 2006). In addition, in mice lacking the CB1R both increased anxiety in the elevated-plus maze test (Haller et al., 2004; Mikics et al., 2009) and reduced anxiolytic action of benzodiazepines have been observed (Urigüen et al., 2004). Conversely, enhancing ECS signaling may exert antidepressant and anxiolytic-like effects. In particular, it has been demonstrated that FAAH inhibitors reduced anxiety-like behavior in the elevated-zero maze and in the isolation-induced ultrasonic-emission tests (Kathuria et al., 2003). Moreover, FAAH inhibitors reduced immobility in the tail suspension test and prolonged swimming in the forced swimming test (Gobbi et al., 2005). In addition, similar results were obtained by administration of either CB1R agonists or an eCB uptake inhibitor (Hill and Gorzalka, 2005b; Adamczyk et al., 2008).

It has also been shown that the clinical effect of different antidepressants is mediated by a modulation of ECS activity in specific brain regions, including the hypothalamus, the amygdala and the hippocampus. In particular, chronic treatment with tricyclic antidepressants was associated with increased CB1R density in the hippocampus and hypothalamus and reduced hypothalamic-pituitary-adrenal (HPA) axis activation in response to stressing stimuli (Hill et al., 2006, 2009a).

It has been evidenced that ECS functionality may influence mood also in humans. In patients with major depression reduced circulating levels of both 2-AG (Hill et al., 2008) and AEA (Hill et al., 2009b) have been described. In addition, chronic treatment with CB1R antagonists has been associated with increased incidence of anxiety and depression. In particular, the CB1R antagonist rimonabant introduced for smoking cessation and to treat obesity, was suspended following several reports of severe mood depressant action (Després et al., 2005; Van Gaal et al., 2005; Padwal and Majumdar, 2007; Traynor, 2007; Rigotti et al., 2009).

Several genetic variants of the genes encoding the cannabis receptor type 1 (CNR1) and FAAH have been identified (Monteleone et al., 2010). CNR1 has been localized to chromosome 6q14-q15 (Hoehe et al., 1991; Zhang et al., 2004). It has been suggested that genetic variability affecting ECS functionality can influence the individual susceptibility to mood disorders (Monteleone et al., 2010). Accordingly, the AAT triplet repeat polymorphism of the CNR1 gene, and different single nucleotide polymorphism (SNPs) of CNR1 and FAAH genes have been associated with depressive symptoms and with major depression (Barrero et al., 2005; Domschke et al., 2008). In major depression, SNPs of the CNR1 may also influence the response to antidepressant treatment (Domschke et al., 2008; Mitjans et al., 2013). Moreover, it has been evidenced that CNR1 SNPs may interact with previous negative experiences increasing the susceptibility to depression (Juhasz et al., 2009). In particular, in patients with major depression, CNR1 SNPs may affect responsiveness of subcortical structures, including the amygdala and striatum, to social rewarding stimuli (Domschke et al., 2008).

Overall, data from animal models and clinical studies suggest that the ECS functionality could influence anxiety and depression. The mechanisms underlying the interaction between ECS and mood are different and include the modulation of activity in limbic areas involved in reward processing, regulation of other neurotransmitters (i.e., noradrenaline, NA; DA; 5-HT), and HPA activation in the stress response (Hill and Patel, 2013; Micale et al., 2013).

### The ECS mediates reward

Mesocorticolimbic DA projections to the limbic system, including the amygdala, hippocampus and orbitofrontal cortex (OFC), are critically involved in mediating reward (Koob and Volkow, 2010). In addition to DA other neurotransmitter, including acetylcholine, opiate peptides, glutamate and GABA, are involved in reward.

The ECS could modulate synaptic transmission in each of the abovementioned brain structures (Sidhpura and Parsons, 2011; Panagis et al., 2014), mediating the hedonic effects of different environmental rewarding stimuli (Trezza et al., 2010; Klein et al., 2012; Silvestri and Di Marzo, 2013). Accordingly, it has been evidenced that in CB1R-KO mice and after blockade of CB1R the rewarding effects of cannabinoids (Ledent et al., 1999), opiates (Ledent et al., 1999; Martin et al., 2000; Cossu et al., 2001), and cocaine (Chaperon et al., 1998) were reduced. Conversely, stimulation of CB1R was associated with relapses of substances abuse (Fattore et al., 2007; Higuera-Matas et al., 2008).

Experimental studies demonstrated that sensitization of CB1R-mediated transmission in the striatum may represent the neurophysiological hallmark of different forms of reward-based behavior. In mice, 1-day treatment with cocaine did not modify the synaptic response to CB agonists in the striatum. Conversely, 7 days of cocaine administration induced conditioned place preference, which is associated with hypersensitivity of striatal GABA synapses to the stimulation of CB1Rs (Centonze et al., 2007). Other environmental rewards were also associated with modulation of striatal DA (Mark et al., 1991; Hajnal et al., 2004; El Rawas et al., 2009), and were associated with upregulation of CB transmission in GABAergic striatal terminals (De Chiara et al., 2010). Accordingly, CB1R blockade reduces motivation for sweet foods, whereas activation of these receptors increases it (Simiand et al., 1998; Cota et al., 2003; Ward and Dykstra, 2005; Mahler et al., 2007).

The ECS may affect reward processing also in humans. Indeed, post-mortem studies evidenced altered expression of different ECS components in the prefrontal cortex of subject with alcohol abuse (Erdozain et al., 2014), moreover, reduced CB1R binding has been evidenced *in vivo* in patients with alcohol dependence (Hirvonen et al., 2013). In addition, SNPs of the CNR1 gene have been associated with drug misuse, alcohol withdrawal, and susceptibility to mood disorders (Barrero et al., 2005; Chen et al., 2008; Domschke et al., 2008; Marcos et al., 2012). In addition, also FAAH SNPs has been correlated to the risk of cannabis and alcohol abuse (Tyndale et al., 2007; Bühler et al., 2014). This evidence suggests that genetic variability in the ECS may influence reward sensitivity, and increase the risk of substance misuse (Parsons and Hurd, 2015).

### ECS and stress

Activation of the hypothalamic-pituitary-adrenal (HPA) axis represents the typical response to stress. While the HPA axis activity could be modulated by the ECS, conversely both stress and glucocorticoids could induce eCBs signaling in brain areas critically involved in behavioral responses, such as the hypothalamus and the amygdala (Hill and McEwen, 2010; Hill and Patel, 2013).

Experimental data suggest that the ECS could be crucially involved in mediating the neurophysiological and behavioral consequences of stress. In fact, stressful events may increase the synthesis of eCBs in the periaqueductal gray (Hohmann et al., 2005) and alter eCBs amount in brain areas associated with reward processing (Patel et al., 2005; Rademacher et al., 2008) thus modulating different responses, including stress-induced analgesia (Hohmann et al., 2005) and sensitivity to natural reward (Rademacher and Hillard, 2007). Notably, other neurotransmitter systems, including cholecystokinin signaling, are involved in the regulation of the behavioral consequences of stress (Kurrikoff et al., 2008).

It has been demonstrated that the behavioral effects of social stress in mice were associated with a selective alteration of the sensitivity of GABA synapses to CB1R activation. In particular, exposure to social stress in mice altered the CB1R-mediated control of GABAergic synaptic transmission in the striatum (Rossi et al., 2008). The specific role of stress is confirmed by the evidence that striatal synaptic alterations are prevented by glucocorticoid receptors blockade and reproduced by glucocorticoids administration. Finally, stress-induced synaptic alterations were rescued after either exposure to natural rewards (i.e., running wheel, sucrose) or administration of cocaine (Rossi et al., 2008). These data suggest that the ECS integrates different environmental stimuli, modulating their effects on striatal synaptic transmission. Notably, different types of rewards administered before the exposure to stress, may exert a protective effect against stress-induced synaptic alterations (De Chiara et al., 2010). In addition, human studies showed that ECS functionality could influence the response to stress (Neumeister et al., 2013; Pietrzak et al., 2014). In particular, different SNPs of the ECS have been associated to post-traumatic stress disorder (PTSD), influencing the effects of previous stressful events (Lu et al., 2008; Agrawal et al., 2012; Mota et al., 2015).

## Inflammation and mood

Recent research has highlighted the relationship between the immune response and mood disturbances. In different inflammatory conditions, proinflammatory mediators could induce anxiety, anhedonia, social withdrawal, fatigue, and sleep disturbances, defined as “sickness behavior” (Raison et al., 2006; Dantzer et al., 2008; Miller et al., 2009). Accordingly, systemic administration of proinflammatory agents is able to promote the activation of microglial cells in the hippocampus and stimulate the release of proinflammatory cytokines in the CNS (van Dam et al., 1992; Breder et al., 1994; Layé et al., 1994; Riazi et al., 2008), and is associated with depression and anxiety (Reichenberg et al., 2001; Miller et al., 2009). Different inflammatory molecules, including interleukin (IL)-1β and tumor necrosis factor (TNF), have been implicated in the behavioral manifestations. In particular, central administration of these cytokines induces sickness behavior in animals (Dantzer et al., 2008; Haji et al., 2012; Rossi et al., 2012).

In addition, it has been proposed that inflammation may be implicated in the pathophysiology of different psychiatric syndromes, including major depression (Maes et al., 1995; Capuron et al., 2002). Accordingly, elevated biomarkers of systemic inflammation, as C-reactive protein, have been associated with depressive symptoms (Morris et al., 2011). It has been evidenced that depressed patients show higher peripheral blood levels of proinflammatory cytokines (Zorrilla et al., 2001; Dowlati et al., 2010; Haapakoski et al., 2015), and elevated inflammatory markers predict reduced response to antidepressant treatment (Strawbridge et al., 2015). Accordingly, increased prevalence of depression has been observed in patients with autoimmune disorders (Zeher et al., 2010), and blocking cytokine signaling can exert beneficial effects on mood (Tyring et al., 2006). Moreover, post-mortem studies in patients with major depression evidenced that also the innate immune response is altered, possibly contributing to the pathogenesis of depression (Martín-Hernández et al., 2018). Finally, it has been suggested that also the efficacy of different psychoactive treatments may be mediated by immunomodulatory properties (Maes et al., 1999; Cattaneo et al., 2013; Horowitz et al., 2015).

The effects of neuroinflammation on mood have been extensively investigated in neurological diseases and particularly in multiple sclerosis (MS). Anxiety and depression, are more frequent in MS patients compared to both general population (Patten et al., 2003) and other neurological patients (Schiffer and Babigian, 1984; Schubert and Foliart, 1993; Thielscher et al., 2013). In particular, mood disturbances may be independent of other neurological deficits and may occur as a presenting symptom (Haussleiter et al., 2009; Lo Fermo et al., 2010; Suh et al., 2010). It has been shown that in animal models of MS (i.e., experimental autoimmune encephalomyelitis, EAE) sickness behavior is observed during the acute phase before the onset of motor signs (Pollak et al., 2000) and comes from altered neuronal activity produced by the inflammatory milieu (Gentile et al., 2015a).

Experimental studies showed that in EAE specific proinflammatory cytokines, particularly TNF and IL-1β, are critically involved in the induction of mood alterations (Pollak et al., 2003; Gentile et al., 2015b). Notably, the synaptic alterations have been found in the striatum, a key structure involved in mood regulation both in rodents and humans (Mathew and Ho, 2006; Nestler and Carlezon, 2006; Kim et al., 2008; Zhang et al., 2008). In particular, striatal synaptic degeneration and dendritic spine loss have been found already during the early phases of EAE, independently of demyelination or clinical disability (Centonze et al., 2009). This data suggest that TNF-induced striatal synaptic dysfunction may be responsible of the behavioral manifestations. In line with these findings, intracerebroventricular injection of TNF in control mice was associated with anxious behavior (Haji et al., 2012). Conversely, intracerebroventricular administration of etanercept, a TNF inhibitor, reduced anxious behavior and prevented the synaptic alteration in EAE mice (Haji et al., 2012). In addition, elevated levels of IL-1β and IL-1β mRNA have been evidenced in the striatum of EAE mice, together with reduced dopaminergic transmission (Gentile et al., 2015b). Accordingly, blocking IL-1β signaling in EAE mice can reduce mood alterations and restore DA release in the striatum (Gentile et al., 2015b).

### Inflammation and ECS

A complex bidirectional interaction exists between the ECS and the immune system. eCBs signaling has immunosuppressant actions, in particular, CB2R stimulation reduce both inflammation (Pandey et al., 2009) and circulating proinflammatory mediators (Croxford and Miller, 2003). Conversely, lipopolysaccharide administration modulates eCB signaling (Klein et al., 2003). In particular, microglial cells are recently emerging as key elements mediating the relationship between inflammation and the ECS. Microglia express CBRs, particularly CB2Rs, and is able to release both inflammatory mediators and eCBs (Stella, 2009; Boorman et al., 2016). Notably, CB2R stimulation in microglial cells promote the release of anti-inflammatory cytokines (Ma et al., 2015). Therefore, reduced CB2R stimulation may contribute to the pathogenesis of mood disorders. Accordingly, it has been evidenced that a polymorphism of the CB2R gene, producing reduced activation of the CB2R receptor, was associated with increased incidence of depression (Onaivi et al., 2008). Moreover, it has been demonstrated that treatment with both minocycline and antidepressant drugs was associated to reduced release of proinflammatory molecules by microglial cells (Burke et al., 2014; Su et al., 2015).

Several studies evidenced that the ECS plays a critical role in mediating the effects of inflammation on synaptic functioning and mood. Accordingly, in EAE mice, CB1Rs stimulation may counteract the TNF-mediated synaptic alterations (Rossi et al., 2011). In particular, administration of a CB1R agonist, prevented the alterations of striatal transmission produced by TNF on brain slices.

Several evidences suggest that specific proinflammatory mediators, including IL-1β and IFNɤ, modulate striatal CB1R sensitivity (Rossi et al., 2012; Gentile et al., 2016; Mandolesi et al., 2017). In particular, intracerebroventricular administration of IL-1β in control mice induced anxious behavior and was associated with reduced sensitivity of striatal GABAergic synapses to CB1Rs stimulation (Rossi et al., 2012). It has been observed that IL-1β mediates the effects of inflammation in EAE mice (Gentile et al., 2016). Accordingly, blocking IL-1β signaling restored striatal CB1Rs sensitivity and reduced anxious behavior (Gentile et al., 2016).

Interestingly, it has been shown that inflammatory signals and environmental stimuli interact with the ECS to produce mood changes. In particular, the effects of IL-1β administration on striatal transmission were similar to the alterations induced by social defeat stress (Rossi et al., 2008). Furthermore, administration of IL-1β receptor antagonist (IL-1ra) was able to revert the synaptic and behavioral effects induced by both IL-1β injection and social defeat stress. These results suggest that IL-1β is involved in inflammation-induced mood alterations and also play a key role in mediating responses to environmental stress (Koo and Duman, 2008; Goshen and Yirmiya, 2009; Norman et al., 2010).

## Therapeutic application of cannabinoids

Cannabis contains numerous active components. In particular, Δ9-tetrahydrocannabinol (THC) and cannabidiol (CBD) represent the main phytocannabinoids (Huestis, 2007). In addition to smoked cannabis, different commercially available compounds have been produced (i.e., dronabinol, nabilone, Sativex^®;^), differing in THC and CBD composition. Cannabis-derived treatments are currently approved for several clinical conditions, including spasticity, pain, nausea, and some epileptic conditions (Pacher and Kunos, 2013; Devinsky et al., 2014). However, despite numerous reports of beneficial effects on anxiety and depression, the clinical efficacy is still debated (Whiting et al., 2015; Turna et al., 2017). In particular, recent reviews and meta-analyses, concluded that evidence supporting clinical benefits of cannabis-based therapies in mood disorders is scarce, and limited to low-grade evidence supporting the beneficial effect of CBD in social anxiety (Bergamaschi et al., 2011) and of medical marijuana in PTSD (Walsh et al., 2017).

The limited approved clinical indications contrast with the ample evidence, derived by preclinical studies and also from anecdotal observations, showing beneficial effects of ECS modulation on mood. It should be considered that a number of factors may contribute to the lack of clear findings. It has been observed that most clinical studies were considered as at high risk of bias, generally for incomplete report of outcome measures and for inadequate blinding procedure (Whiting et al., 2015). In addition, clinical studies mainly explored the effects of synthetic principles, and studies exploring the effects of cannabis are limited. Furthermore, the role of genetic individual variability should be considered, as differences in CB1Rs sensitivity to phytocannabinoids may explain the ample variability of the effects reported by patients.

A specific confounding factor that limits the clinical evidences supporting the beneficial effects of cannabinoids on mood is the difficulty in controlling the placebo effect. It is worth noting that the placebo response could significantly influence the clinical effect of drugs acting on the ECS (Benedetti et al., 2011a; Di Marzo and Centonze, 2015). Placebo effect constitutes a complex phenomenon coming from both the social ritual represented by placebo administration and the psychosocial context (Benedetti et al., 2011a). Interestingly, it has been clearly demonstrated that both the placebo response and drugs activate the same biochemical pathways. In particular, the placebo analgesia is mediated by both the opioid system and the ECS (Wager et al., 2007; Benedetti et al., 2011b). Consequently, it has been demonstrated that the administration of rimonabant inhibits the non-opioid placebo response (Benedetti et al., 2011b). In clinical trials evaluating the efficacy of antidepressant medications, it has been shown that the placebo effect could be responsible for at least one-half of the beneficial effect (Kirsch and Sapirstein, 1998) representing a relevant confounding factor. Indeed, as ECS mediates both the antidepressant effect of drugs and the placebo response, patients responding to antidepressant drugs may also show a marked placebo response.

Finally, concerns about the risks associated to cannabis use have limited the clinical indications of medical marijuana. In particular, acute cannabis use has also been associated with worsening of anxiety (Crippa et al., 2009). Furthermore, it has been proposed that chronic consumption could be associated with increased risk of psychosis, cognitive impairment and addictiveness in predisposed subjects (Volkow et al., 2014; Ksir and Hart, 2016; Turna et al., 2017).

## Conclusions

Emotional homeostasis is crucially modulated by the activity of the ECS. In particular, different environmental and endogenous stimuli could influence the emotional state by modulating the sensitivity to eCBs of different neurotransmitter pathways in multiple brain areas. Cannabis-based compounds could exert antidepressant effects through complex influences on different behavioral responses, such as those associated to reward, stress and inflammation, also depending on the individual psychosocial context. Overall, these issues make it difficult to demonstrate unequivocal relationships between ECS modulation and the effects on mood. Although targeting the ECS could represent a promising treatment option in different psychiatric conditions, future clinical trials should be designed to explore specific outcome measures, to reduce the individual variability and to consider the placebo response. In the last years, the ample diffusion of smoked or vaporized cannabis for recreational and therapeutic purposes has not been accompanied by measures aimed at promoting information about medical marijuana use. In this view, it is important to design specific interventions to overcome the gap between preclinical studies and clinical evidences on the potential therapeutic use of cannabinoids.

## Author contributions

MS and EI: work conception and design, drafting the work, work revision, final approval, and global agreement. LG: work conception and design, work revision, final approval, and global agreement. PM, ED, AB, and FB: work revision, final approval, and global agreement. DC: work conception and design, guarantor of integrity of entire study, manuscript revision for important intellectual content, final approval.

### Conflict of interest statement

The authors declare that the research was conducted in the absence of any commercial or financial relationships that could be construed as a potential conflict of interest.

## References

[B1] Adamczyk P. Gołda A. McCreary A. C. Filip M. Przegalinski E. (2008). Activation of endocannabinoid transmission induces antidepressant-like effects in rats. J. Physiol. Pharmacol. 59, 217–228. 18622041

[B2] Agrawal A. Nelson E. C. Littlefield A. K. Bucholz K. K. Degenhardt L. Henders A. K. (2012). Cannabinoid receptor (CNR1) genotype moderates the effects of childhood physical abuse on anhedonia and depression. Arch. Gen. Psychiatry 69, 732–740. 10.1001/archgenpsychiatry.2011.2273 22393204 PMC3706194

[B3] Alger B. E. (2002). Retrograde signaling in the regulation of synaptic transmission: focus on endocannabinoids. Prog. Neurobiol. 68, 247–286. 10.1016/S0301-0082(02)00080-1 12498988

[B4] Barrero F. J. Ampuero I. Morales B. Vives F. de Dios Luna Del Castillo J. Hoenicka J.. (2005). Depression in Parkinson's disease is related to a genetic polymorphism of the cannabinoid receptor gene (CNR1). Pharmacogenom. J. 5, 135–141. 10.1038/sj.tpj.6500301 15668727

[B5] Benedetti F. Amanzio M. Rosato R. Blanchard C. (2011b). Nonopioid placebo analgesia is mediated by CB1 cannabinoid receptors. Nat. Med. 17, 1228–1230. 10.1038/nm.2435 21963514

[B6] Benedetti F. Carlino E. Pollo A. (2011a). How placebos change the patient's brain. Neuropsychopharmacology. 36, 339–354. 10.1038/npp.2010.81 20592717 PMC3055515

[B7] Bergamaschi M. M. Queiroz R. H. Zuardi A. W. Crippa J. A. (2011). Safety and side effects of cannabidiol, a Cannabis sativa constituent. Curr. Drug Saf. 6, 237–249. 10.2174/157488611798280924 22129319

[B8] Boorman E. Zajkowska Z. Ahmed R. Pariante C. M. Zunszain P. A. (2016). Crosstalk between endocannabinoid and immune systems: a potential dysregulation in depression? Psychopharmacology 233, 1591–1604. 10.1007/s00213-015-4105-9 26483037 PMC4828487

[B9] Breder C. D. Hazuka C. Ghayur T. Klug C. Huginin M. Yasuda K.. (1994). Regional induction of tumor necrosis factor alpha expression in the mouse brain after systemic lipopolysaccharide administration. Proc. Natl. Acad. Sci. U.S.A. 91, 11393–11397. 10.1073/pnas.91.24.11393 7972071 PMC45237

[B10] Bühler K. M. Huertas E. Echeverry-Alzate V. Ginè E. Moltó E. Montoliu L.. (2014). Risky alcohol consumption in young people is associated with the fatty acid amide hydrolase gene polymorphism C385A and affective rating of drug pictures. Mol. Genet. Genomics. 289, 279–289. 10.1007/s00438-013-0809-x 24407958

[B11] Burke N. N. Kerr D. M. Moriarty O. Finn D. P. Roche M. (2014). Minocycline modulates neuropathic pain behaviour and cortical M1-M2 microglial gene expression in a rat model of depression. Brain Behav. Immun. 42, 147–156. 10.1016/j.bbi.2014.06.015 24994592

[B12] Capuron L. Hauser P. Hinze-Selch D. Miller A. H. Neveu P. J. (2002). Treatment of cytokine-induced depression. Brain Behav. Immun. 16, 575–580. 10.1016/S0889-1591(02)00007-7 12401471

[B13] Castillo P. E. Younts T. J. Chávez A. E. Hashimotodani Y. (2012). Endocannabinoid signaling and synaptic function. Neuron 76, 70–81. 10.1016/j.neuron.2012.09.020 23040807 PMC3517813

[B14] Cattaneo A. Gennarelli M. Uher R. Breen G. Farmer A. Aitchison K. J.. (2013). Candidate genes expression profile associated with antidepressants response in the GENDEP study: differentiating between baseline ‘predictors' and longitudinal ‘targets'. Neuropsychopharmacology 38, 377–385. 10.1038/npp.2012.191 22990943 PMC3547188

[B15] Centonze D. Muzio L. Rossi S. Cavasinni F. De Chiara V. Bergami A.. (2009). Inflammation triggers synaptic alteration and degeneration in experimental autoimmune encephalomyelitis. J. Neurosci. 29, 3442–3452. 10.1523/JNEUROSCI.5804-08.2009 19295150 PMC6665268

[B16] Centonze D. Rossi S. De Chiara V. Prosperetti C. Battista N. Bernardi G.. (2007). Chronic cocaine sensitizes striatal GABAergic synapses to the stimulation of cannabinoid CB1 receptors. Eur. J. Neurosci. 25, 1631–1640. 10.1111/j.1460-9568.2007.05433.x 17408430

[B17] Chaperon F. Soubrié P. Puech A. J. Thiébot M. H. (1998). Involvement of central cannabinoid (CB1) receptors in the establishment of place conditioning in rats. Psychopharmacology 135, 324–332. 9539255 10.1007/s002130050518

[B18] Chen X. Williamson V. S. An S.-S. Hettema J. M. Aggen S. H. Neale M. C.. (2008). Cannabinoid receptor 1 gene association with nicotine dependence. Arch. Gen. Psychiatry 65, 816–824. 10.1001/archpsyc.65.7.816 18606954 PMC2733353

[B19] Chiang N. Dalli J. Colas R. A. Serhan C. N. (2015). Identification of resolvin D2 receptor mediating resolution of infections and organ protection. J. Exp. Med. 212, 1203–1217. 10.1084/jem.20150225 26195725 PMC4516788

[B20] Cossu G. Ledent C. Fattore L. Imperato A. Bohme G. A. Parmentier M. (2001). Cannabinoid CB1 receptor knockout mice fail to selfadminister morphine but not other drugs of abuse. Behav. Brain. Res. 118, 61–65. 10.1016/S0166-4328(00)00311-9 11163634

[B21] Cota D. Marsicano G. Lutz B. Vicennati V. Stalla G. K. Pasquali R.. (2003). Endogenous cannabinoid system as a modulator of food intake. Int. J. Obes. Relat. Metab. Disord. 27, 289–301. 10.1038/sj.ijo.802250 12629555

[B22] Crippa J. A. Zuardi A. W. Martín-Santos R. Bhattacharyya S. Atakan Z. McGuire P.. (2009). Cannabis and anxiety: a critical review of the evidence. Hum. Psychopharmacol. 24, 515–523. 10.1002/hup.1048 19693792

[B23] Croxford J. L. Miller S. D. (2003). Immunoregulation of a viral model of multiple sclerosis using the synthetic cannabinoid R+WIN55,212. Clin Invest. 111, 1231–1240. 10.1172/JCI17652 12697742 PMC152941

[B24] Dantzer R. O'Connor J. C. Freund G. G. Johnson R. W. Kelley K. W. (2008). From inflammation to sickness and depression: when the immune system subjugates the brain. Nat. Rev. Neurosci. 9, 46–56. 10.1038/nrn2297 18073775 PMC2919277

[B25] De Chiara V. Errico F. Musella A. Rossi S. Mataluni G. Sacchetti L.. (2010). Voluntary exercise and sucrose consumption enhance cannabinoid CB1 receptor sensitivity in the striatum. Neuropsychopharmacology 35, 374–387. 10.1038/npp.2009.141 19776732 PMC3055381

[B26] Després J. P. Golay A. Sjöström L. for the Rimonabant in Obesity-Lipids Study Group. (2005). Effects of rimonabant on metabolic risk factors in overweight patients with dyslipidemia. N. Engl. J. Med. 353, 2121–2134. 10.1056/NEJMoa04453 16291982

[B27] Devinsky O. Cilio M. R. Cross H. Fernandez-Ruiz J. French J. Hill C.. (2014). Cannabidiol: pharmacology and potential therapeutic role in epilepsy and other neuropsychiatric disorders. Epilepsia 55, 791–802. 10.1111/epi.12631 24854329 PMC4707667

[B28] Di Marzo V. (2009). The endocannabinoid system: its general strategy of action, tools for its pharmacological manipulation and potential therapeutic exploitation. Pharmacol. Res. 60, 77–84. 10.1016/j.phrs.2009.02.010 19559360

[B29] Di Marzo V. Centonze D. (2015). Placebo effects in a multiple sclerosis spasticity enriched clinical trial with the oromucosal cannabinoid spray (THC/CBD): dimension and possible causes. CNS Neurosci Ther. 21, 215–221. 10.1111/cns.12358 25475413 PMC6495119

[B30] Di Marzo V. Melck D. Bisogno T. De Petrocellis L. (1998). Endocannabinoids: endogenous cannabinoid receptor ligands with neuromodulatory action. Trends Neurosci. 21, 521–528. 10.1016/S0166-2236(98)01283-1 9881850

[B31] Domschke K. Dannlowski U. Ohrmann P. Lawford B. Bauer J. Kugel H.. (2008). Cannabinoid receptor 1 (CNR1) gene: impact on antidepressant treatment response and emotion processing in major depression. Eur. Neuropsychopharmacol. 18, 751–759. 10.1016/j.euroneuro.2008.05.003 18579347

[B32] Dowlati Y. Herrmann N. Swardfager W. Liu H. Sham L. Reim E. K.. (2010). A meta-analysis of cytokines in major depression. Biol. Psichiatry 67, 446–457. 10.1016/j.biopsych.2009.09.033 20015486

[B33] El Rawas R. Thiriet N. Lardeux V. Jaber M. Solinas M. (2009). Environmental enrichment decreases the rewarding but not the activating effects of heroin. Psychopharmacology 203, 561–570. 10.1007/s00213-008-1402-6 19005643

[B34] Erdozain A. M. Rubio M. Valdizan E. M. Pazos A. Meana J. J. Fernández-Ruiz J.. (2014). The endocannabinoid system is altered in the post-mortem prefrontal cortex of alcoholic subjects. Addict. Biol. 20, 773–783. 10.1111/adb.12160 25041461

[B35] Farran B. (2017). An update on the physiological and therapeutic relevance of GPCR oligomers. Pharmacol. Res. 117, 303–327. 10.1016/j.phrs.2017.01.008 28087443

[B36] Fattore L. Spano M. S. Deiana S. Melis V. Cossu G. Fadda P.. (2007). An endocannabinoid mechanism in relapse to drug seeking: a review of animal studies and clinical perspectives. Brain Res. Rev. 53, 1–16. 10.1016/j.brainresrev.2006.05.003 16839608

[B37] Gentile A. De Vito F. Fresegna D. Musella A. Buttari F. Bullitta S.. (2015a). Exploring the role of microglia in mood disorders associated with experimental multiple sclerosis. Front. Cell. Neurosci. 9:243. 10.3389/fncel.2015.00243 26161070 PMC4479791

[B38] Gentile A. Fresegna D. Federici M. Musella A. Rizzo F. R. Sepman H.. (2015b). Dopaminergic dysfunction is associated with IL-1β-dependent mood alterations in experimental autoimmune encephalomyelitis. Neurobiol. Dis. 74, 347–358. 10.1016/j.nbd.2014.11.022 25511803

[B39] Gentile A. Fresegna D. Musella A. Sepman H. Bullitta S. De Vito F.. (2016). Interaction between interleukin-1β and type-1 cannabinoid receptor is involved in anxiety-like behavior in experimental autoimmune encephalomyelitis. J. Neuroinflammation 13, 231. 10.1186/s12974-016-0682-8. 27589957 PMC5009553

[B40] Glass M. Dragunow M. Faull R. L. (1997). Cannabinoid receptors in the human brain: a detailed anatomical and quantitative autoradiographic study in the fetal, neonatal and adult human brain. Neuroscience 77, 299–318. 10.1016/S0306-4522(96)00428-9 9472392

[B41] Gobbi G. Bambico F. R. Mangieri R. Bortolato M. Campolongo P. Solinas M.. (2005). Antidepressant-like activity and modulation of brain monoaminergic transmission by blockade of anandamide hydrolysis. Proc. Natl. Acad. Sci. U.S.A. 102, 18620–18625. 10.1073/pnas.0509591102 16352709 PMC1317988

[B42] Goshen I. Yirmiya R. (2009). Interleukin-1 (IL-1): a central regulator of stress responses. Front. Neuroendocrinol. 30, 30–45. 10.1016/j.yfrne.2008.10.001 19017533

[B43] Haapakoski R. Mathieu J. Ebmeier K. P. Alenius H. Kivimäki M. (2015). Cumulative meta-analysis of interleukins 6 and 1β, tumour necrosis factor α and C-reactive protein in patients with major depressive disorder. Brain Behav. Immun. 49, 206–215. 10.1016/j.bbi.2015.06.001 26065825 PMC4566946

[B44] Haji N. Mandolesi G. Gentile A. Sacchetti L. Fresegna D. Rossi S.. (2012). TNF-α-mediated anxiety in a mouse model of multiple sclerosis. Exp. Neurol. 237, 296–303. 10.1016/j.expneurol.2012.07.010 22836148

[B45] Hajnal A. Smith G. P. Norgren R. (2004). Oral sucrose stimulation increases accumbens dopamine in the rat. Am. J. Physiol. Regul. Integr. Comp. Physiol. 286, 31–37. 10.1152/ajpregu.00282.2003 12933362

[B46] Haller J. Varga B. Ledent C. Freund T. F. (2004). CB1 cannabinoid receptors mediate anxiolytic effects: convergent genetic and pharmacological evidence with CB1-specific agents. Behav. Pharmacol. 15, 299–304. 10.1097/01.fbp.0000135704.56422.40 15252281

[B47] Haussleiter I. S. Brüne M. Juckel G. (2009). Review: Psychopathology in multiple sclerosis: diagnosis, prevalence and treatment. Ther. Adv. Neurol. Disord. 2, 13–29. 10.1177/1756285608100325 21180640 PMC3002616

[B48] Heifets B. D. Castillo P. E. (2009). Endocannabinoid signaling and long-term synaptic plasticity. Ann. Rev. Physiol. 71, 283–306. 10.1146/annurev.physiol.010908.163149 19575681 PMC4454279

[B49] Heinbockel T. Brager D. H. Reich C. G. Zhao J. Muralidharan S. Alger B. E.. (2005). Endocannabinoid signaling dynamics probed with optical tools. J. Neurosci. 25, 9449–9459. 10.1523/JNEUROSCI.2078-05.2005 16221855 PMC6725697

[B50] Herkenham M. Lynn A. B. Johnson M. R. Melvin L. S. de Costa B. R. Rice K. C. (1991). Characterization and localization of cannabinoid receptors in rat brain: a quantitative *in vitro* autoradiographic study. J. Neurosci. 11, 563–583. 10.1523/JNEUROSCI.11-02-00563.1991 1992016 PMC6575215

[B51] Higuera-Matas A. Soto-Montenegro M. L. Del Olmo N. Miguens M. Torres I. Vaquero J. J. (2008). Augmented acquisition of cocaine self-administration and altered brain glucose metabolism in adult female but not male rats exposed to a cannabinoid agonist during adolescence. Neuropsychopharmacology 33, 806–813. 10.1038/sj.npp.1301467 17551541

[B52] Hill M. N. Gorzalka B. B. (2005a). Is there a role for the endocannabinoid system in the etiology and treatment of melancholic depression? Behav. Pharmacol. 16, 333–352. 16148438 10.1097/00008877-200509000-00006

[B53] Hill M. N. Gorzalka B. B. (2005b). Pharmacological enhancement of cannabinoid CB1 receptor activity elicits an antidepressant-like response in the rat forced swim test. Eur. Neuropsychopharmacol. 15, 593–599. 10.1016/j.euroneuro.2005.03.003 15916883

[B54] Hill M. N. Hillard C. J. Bambico F. R. Patel S. Gorzalka B. B. Gobbi G. (2009a). The therapeutic potential of the endocannabinoid system for the development of a novel class of antidepressants. Trends Pharmacol. Sci. 30, 484–493. 10.1016/j.tips.2009.06.006 19732971

[B55] Hill M. N. Ho W. S. Sinopoli K. J. Viau V. Hillard C. J. Gorzalka B. B. (2006). Involvement of the endocannabinoid system in the ability of long-term tricyclic antidepressant treatment to suppress stress-induced activation of the hypothalamic-pituitary-adrenal axis. Neuropsychopharmacology 31, 2591–2599. 10.1038/sj.npp.1301092 16710317

[B56] Hill M. N. McEwen B. S. (2010). Involvement of the endocannabinoid system in the neurobehavioural effects of stress and glucocorticoids. Prog. Neuropsychopharmacol. Biol. Psychiatry 34, 791–797. 10.1016/j.pnpbp.2009.11.001 19903506 PMC2945244

[B57] Hill M. N. Miller G. E. Carrier E. J. Gorzalka B. B. Hillard C. J. (2009b). Circulating endocannabinoids and N-acyl ethanolamines are differentially regulated in major depression and following exposure to social stress. Psychoneuroendocrinology 34, 1257–1262. 10.1016/j.psyneuen.2009.03.013 19394765 PMC2716432

[B58] Hill M. N. Miller G. E. Ho W. S. Gorzalka B. B. Hillard C. J. (2008). Serum endocannabinoid content is altered in females with depressive disorders: a preliminary report. Pharmacopsychiatry 41, 48–53. 10.1055/s-2007-993211 18311684 PMC3422568

[B59] Hill M. N. Patel S. (2013). Translational evidence for the involvement of the endocannabinoid system in stress-related psychiatric illnesses. Biol. Mood Anxiety Disord. 3, 1–19. 10.1186/2045-5380-3-19 24286185 PMC3817535

[B60] Hirvonen J. Zanotti-Fregonara P. Umhau J. C. George D. T. Rallis-Frutos D. Lyoo C. H.. (2013). Reduced cannabinoid CB1 receptor binding in alcohol dependence measured with positron emission tomography. Mol. Psychiatry 18, 916–921. 10.1038/mp.2012.100 22776901 PMC3594469

[B61] Hoehe M. R. Caenazzo L. Martinez M. M. Hsieh W. T. Modi W. S. Gershon E. S.. (1991). Genetic and physical mapping of the human cannabinoid receptor gene to chromosome 6q14-q15. New Biol. 3, 880–885. 1931832

[B62] Hohmann A. G. Suplita R. L. Bolton N. M. Neely M. H. Fegley D. Mangieri R.. (2005). An endocannabinoid mechanism for stress-induced analgesia. Nature 435, 1108–1112. 10.1038/nature03658 15973410

[B63] Horowitz M. A. Wertz J. Zhu D. Cattaneo A. Musaelyan K. Nikkheslat N.. (2015). Antidepressant compounds can be both pro- and anti-inflammatory in human hippocampal cells. Int. J. Neuropsychopharmacol. 18, 3. 10.1093/ijnp/pyu076. 25522414 PMC4360247

[B64] Howlett A. C. Barth F. Bonner T. I. Cabral G. Casellas W. A. Devane C. C.. (2002). International Union of Pharmacology. XXVII. Classification of cannabinoid receptors. Pharmacol. Rev. 54, 161–202. 10.1124/pr.54.2.161 12037135

[B65] Huestis M. A. (2007). Human cannabinoid pharmacokinetics. Chem. Biodivers. 4, 1770–1804. 10.1002/cbdv.200790152 17712819 PMC2689518

[B66] Juhasz G. Chase D. Pegg E. Downey D. Toth Z. G. Stones K.. (2009). CNR1 gene is associated with high neuroticism and low agreeableness and interacts with recent negative life events to predict current depressive symptoms. Neuropsychopharmacology 34, 2019–2027. 10.1038/npp.2009.19 19242408

[B67] Kano M. Ohno-Shosaku T. Hashimotodani Y. Uchigashima M. Watanabe M. (2009). Endocannabinoid-mediated control of synaptic transmission. Physiol. Rev. 89, 309–380. 10.1152/physrev.00019.2008 19126760

[B68] Kathuria S. Gaetani S. Fegley D. Valiño F. Duranti A. Tontini A.. (2003). Modulation of anxiety through blockade of anandamide hydrolysis. Nat. Med. 9, 76–81. 10.1038/nm803 12461523

[B69] Kim K. S. Lee K. W. Baek I. S. Lim C. M. Krishnan V. Lee J. K.. (2008). Adenylyl cyclase-5 activity in the nucleus accumbens regulates anxiety-related behavior. J. Neurochem. 107, 105–115. 10.1111/j.1471-4159.2008.05592.x 18673448 PMC2744302

[B70] Kirsch I. Sapirstein G. (1998). Listening to Prozac but hearing placebo: a meta-analysis of antidepressant medication. Prevent. Treat. 1:2a 10.1037/1522-3736.1.1.12a

[B71] Klein C. Hill M. N. Chang S. C. Hillard C. J. Gorzalka B. B. (2012). Circulating endocannabinoid concentrations and sexual arousal in women. J. Sex. Med. 9, 1588–1601. 10.1111/j.1743-6109.2012.02708.x 22462722 PMC3856894

[B72] Klein T. W. Newton C. Larsen K. Lu L. Perkins I. Nong L.. (2003). The cannabinoid system and immune modulation. J. Leukoc. Biol. 74, 486–496. 10.1189/jlb.0303101 12960289

[B73] Koo J. W. Duman R. S. (2008). IL-1beta is an essential mediator of the antineurogenic and anhedonic effects of stress. Proc. Natl. Acad. Sci. U.S.A. 105, 751–756. 10.1073/pnas.0708092105 18178625 PMC2206608

[B74] Koob G. F. Volkow N. D. (2010). Neurocircuitry of addiction. Neuropsychopharmacology 35, 217–238. 10.1038/npp.2009.110 19710631 PMC2805560

[B75] Ksir C. Hart C. L. (2016). Cannabis and psychosis: a critical overview of the relationship. Curr. Psychiatry Rep. 18, 12. 10.1007/s11920-015-0657-y 26781550

[B76] Kurrikoff K. Inno J. Matsui T. Vasar E. (2008). Stress-induced analgesia in mice: evidence for interaction between endocannabinoids and cholecystokinin. Eur. J. Neurosci. 27, 2147–2155. 10.1111/j.1460-9568.2008.06160.x 18412633

[B77] Layé S. Parnet P. Goujon E. Dantzer R. (1994). Peripheral administration of lipopolysaccharide induces the expression of cytokine transcripts in the brain and pituitary of mice. Mol. Brain Res. 27, 157–162. 10.1016/0169-328X(94)90197-X 7877446

[B78] Ledent C. Valverde O. Cossu G. Petitet F. Aubert J. F. Beslot F.. (1999). Unresponsiveness to cannabinoids and reduced addictive effects of opiates in CB1 receptor knockout mice. Science 283, 401–404. 10.1126/science.283.5400.401 9888857

[B79] Lo Fermo S. Barone R. Patti F. Laisa P. Cavallaro T. L. Nicoletti A.. (2010). Outcome of psychiatric symptoms presenting at onset of multiple sclerosis: a retrospective study. Mult. Scler. 16, 742–748. 10.1177/1352458510365157 20350959

[B80] Lovinger D. M. (2008). Presynaptic modulation by endocannabinoids, in: Pharmacology of Neurotransmitter Release. Handbook of Experimental Pharmacology (Berlin: Springer, Heidelberg), 435–477. 10.1007/978-3-540-74805-2_14 18064422

[B81] Lu A. T. Ogdie M. N. Jarvelin M.-R. Moilanem I. K. Loo S. K. McCracken J. T. (2008). Association of the cannabinoid receptor gene (CNR1) with ADHD and post-traumatic stress disorder. Am. J. Med. Genet. B Neuropsychiatr. Genet. 8, 1488–1494. 10.1002/ajmg.b.30693 PMC2685476 18213623

[B82] Lutz B. Marsicano G. Maldonado R. Hillard C. J. (2015). The endocannabinoid system in guarding against fear, anxiety and stress. Nat. Rev. Neurosci. 16, 705–718. 10.1038/nrn4036 26585799 PMC5871913

[B83] Ma L. Jia J. Liu X. Bai F. Wang Q. Xiong L. (2015). Activation of murine microglial N9 cells is attenuated through cannabinoid receptor CB2 signaling. Biochem. Biophys. Res. Commun. 458, 92–97. 10.1016/j.bbrc.2015.01.073 25637536

[B84] Maes M. Meltzer H. Y. Bosmans E. Bergmans R. Vandoolaeghe E. Ranjan R.. (1995). Increased plasma concentrations of interleukin-6, soluble interleukin-6, soluble interleukin-2 and transferrin receptor in major depression. J. Affect. Disord. 34, 301–309. 10.1016/0165-0327(95)00028-L 8550956

[B85] Maes M. Song C. Lin A.-H. Bonaccorso S. Kenis G. De Jongh R.. (1999). Negative immunoregulatory effects of antidepressants: inhibition of interferon-gamma and stimulation of interleukin-10 secretion. Neuropsychopharmacology 20, 370–379. 10.1016/S0893-133X(98)00088-8 10088138

[B86] Mahler S. V. Smith K. S. Berridge K. C. (2007). Endocannabinoid hedonic hotspot for sensory pleasure: anandamide in nucleus accumbens shell enhances ‘liking' of a sweet reward. Neuropsychopharmacology 32, 2267–2278. 10.1038/sj.npp.1301376 17406653

[B87] Mandolesi G. Bullitta S. Fresegna D. Gentile A. De Vito F. Dolcetti E.. (2017). Interferon-γ causes mood abnormalities by altering cannabinoid CB1 receptor function in the mouse striatum. Neurobiol. Dis. 108, 45–53. 10.1016/j.nbd.2017.07.019. 28757328

[B88] Marcos M. Pastor I. de la Calle C. Barrio-Real L. Laso F. J. González-Sarmiento R. (2012). Cannabinoid receptor 1 gene is associated with alcohol dependence. Alcohol. Clin. Exp. Res. 36, 267–271. 10.1111/j.1530-0277.2011.01623.x 22085192

[B89] Mark G. P. Blander D. S. Hoebel B. G. (1991). A conditioned stimulus decreases extracellular dopamine in the nucleus accumbens after the development of a learned taste aversion. Brain Res. 551, 308–310. 10.1016/0006-8993(91)90946-s 1913157

[B90] Martin M. Ledent C. Parmentier M. Maldonado R. Valverde O. (2000). Cocaine, but not morphine, induces conditioned place preference and sensitization to locomotor responses in CB1 knockout mice. Eur. J. Neurosci. 12, 4038–4046. 10.1046/j.1460-9568.2000.00287.x 11069600

[B91] Martin M. Ledent C. Parmentier M. Maldonado R. Valverde O. (2002). Involvement of CB1 cannabinoid receptors in emotional behaviour. Psychopharmacology 159, 379–387. 10.1007/s00213-001-0946-5 11823890

[B92] Martín-Hernández D. Caso J. R. Meana J. J. Callado L. F. Madrigal J. L. M. García-Bueno B.. (2018). Intracellular inflammatory and antioxidant pathways in postmortem frontal cortex of subjects with major depression: effect of antidepressants. J. Neuroinflammation 15, 251. 10.1186/s12974-018-1294-2 30180869 PMC6122627

[B93] Mathew S. J. Ho S. (2006). Etiology and neurobiology of social anxiety disorder. J. Clin. Psychiatry 67, 9–13. 17092190

[B94] Micale V. Di Marzo V. Sulcova A. Wotjak C. T. Drago F. (2013). Endocannabinoid system and mood disorders: priming a target for new therapies. Pharmacol. Ther. 138, 18–37. 10.1016/j.pharmthera.2012.12.002 23261685

[B95] Mikics E. Vas J. Aliczki M. Halasz J. Haller J. (2009). Interactions between the anxiogenic effects of CB1 gene disruption and 5-HT3 neurotransmission. Behav. Pharmacol. 20, 265–272. 10.1097/FBP.0b013e32832c70b1 19421027

[B96] Miller A. H. Maletic V. Raison C. L. (2009). Inflammation and its discontents: the role of cytokines in the pathophysiology of major depression. Biol. Psychiatry 65, 732–741. 10.1016/j.biopsych.2008.11.029 19150053 PMC2680424

[B97] Mitjans M. Serretti A. Fabbri C. Gastó C. Catalán R. Fañanás L.. (2013). Screening genetic variability at the CNR1 gene in both major depression etiology and clinical response to citalopram treatment. Psychopharmacology 227, 509–519. 10.1007/s00213-013-2995-y 23407780

[B98] Monteleone P. Bifulco M. Maina G. Tortorella A. Gazzerro P. Proto M. C.. (2010). Investigation of CNR1 and FAAH endocannabinoid gene polymorphisms in bipolar disorder and major depression. Pharmacol. Res. 61, 400–404. 10.1016/j.phrs.2010.01.002 20080186

[B99] Morales P. Reggio P. H. (2017). An Update on Non-CB_1_, Non-CB_2_ Cannabinoid Related G-Protein-Coupled Receptors. Cannabis Cannabinoid Res. 2, 265–273. 10.1089/can.2017.0036 29098189 PMC5665501

[B100] Morris A. A. Zhao L. Ahmed Y. Stoyanova N. De Staercke C. Hooper W. C.. (2011). Association between depression and inflammation–differences by race and sex: The META-Health Study. Psychosom. Med. 73, 462–468. 10.1097/PSY.0b013e318222379c 21715300 PMC3951048

[B101] Mota N. Sumner J. A. Lowe S. R. Neumeister A. Uddin M. Aiello A. E.. (2015). The rs1049353 polymorphism in the CNR1 gene interacts with childhood abuse to predict posttraumatic threat symptoms. J. Clin. Psychiatry 76, e1622–e1623. 10.4088/JCP.15l10084 26717543 PMC4783167

[B102] Nakazi M. Bauer U. Nickel T. Kathmann M. Schlickler E. (2000). Inhibition of serotonin release in the mouse brain via presynaptic cannabinoid CB1 receptors. Naunyn-Schmiedebergs Arch. Pharmacol. 361, 19–24. 10.1007/s002109900147 10651142

[B103] Navarro M. Hernández E. Muñoz R. M. Del Arco I. Villanua M. A. Carrera M. R.. (1997). Acute administration of the CB1 cannabinoid receptor antagonist SR 141716A induces anxiety-like responses in the rat. NeuroReport 8, 491–496. 10.1097/00001756-199701200-00023 9080435

[B104] Nestler E. J. Carlezon W. A. Jr. (2006). The mesolimbic dopamine reward circuit in depression. Biol. Psychiatry 59, 1151–1159. 10.1016/j.biopsych.2005.09.018 16566899

[B105] Neumeister A. Normandin M. D. Pietrzak R. H. Piomelli D. Zheng M. Q.. (2013). Elevated brain cannabinoid CB1 receptor availability in post-traumatic stress disorder: a positron emission tomography study. Mol. Psychiatry 18, 1034–1040. 10.1038/mp.2013.61 23670490 PMC3752332

[B106] Norman G. J. Karelina K. Zhang N. Walton J. C. Morris J. S. Devries A. C. (2010). Stress and IL-1beta contribute to the development of depressive-like behavior following peripheral nerve injury. Mol. Psychiatry 15, 404–414. 10.1038/mp.2009.91 19773812 PMC5214062

[B107] Onaivi E. S. Ishiguro H. Gong J. P. Patel S. Meozzi P. A. Myers L.. (2008). Brain neuronal CB2 cannabinoid receptors in drug abuse and depression: from mice to human subjects. PLoS ONE 3:e1640. 10.1371/journal.pone.0001640 18286196 PMC2241668

[B108] Pacher P. Kunos G. (2013). Modulating the endocannabinoid system in human health and disease-successes and failures. FEBS J. 280, 1918–1943. 10.1111/febs.12260 23551849 PMC3684164

[B109] Padwal R. S. Majumdar S. R. (2007). Drug treatments for obesity: orlistat, sibutramine, and rimonabant. Lancet 369, 71–77. 10.1016/s0140-6736(07)60033-6 17208644

[B110] Panagis G. Mackey B. Vlachou S. (2014). Cannabinoid regulation of brain reward processing with an emphasis on the role of CB1 receptors: a step back into the future. Front. Psychiatry 5:92. 10.3389/fpsyt.2014.00092 25132823 PMC4117180

[B111] Pandey R. Mousawy K. Nagarkatti M. Nagarkatti P. (2009). Endocannabinoids and immune regulation. Pharmacol. Res. 60, 85–92. 10.1016/j.phrs.2009.03.019 19428268 PMC3044336

[B112] Parsons L. H. Hurd Y. L. (2015). Endocannabinoid signalling in reward and addiction. Nat. Rev. Neurosci. 16, 579–594. 10.1038/nrn4004 26373473 PMC4652927

[B113] Patel S. Hillard C. J. (2006). Pharmacological evaluation of cannabinoid receptor ligands in a mouse model of anxiety: further evidence for an anxiolytic role for endogenous cannabinoid signaling. J. Pharmacol. Exp. Ther. 318, 304–311. 10.1124/jpet.106.101287 16569753

[B114] Patel S. Roelke C. T. Rademacher D. J. Hillard C. J. (2005). Inhibition of restraint stress-induced neural and behavioural activation by endogenous cannabinoid signalling. Eur. J. Neurosci. 21, 1057–1069. 10.1111/j.1460-9568.2005.03916.x 15787710

[B115] Patten S. B. Beck C. A. Williams J. V. Barbui C. Metz L. M. (2003). Major depression in multiple sclerosis: a population-based perspective. Neurology 61, 1524–1527. 10.1212/01.wnl.0000095964.34294.b4 14663036

[B116] Pietrzak R. H. Huang Y. Corsi-Travali S. Zheng M.-Q. Lin S.-F. Henry S.-N.. (2014). Cannabinoid type I receptor availability in the amygdala mediates threat processing in trauma survivors. Neuropsychopharmacology 39, 2519–2528. 10.1038/npp.2014.110 24820537 PMC4207337

[B117] Pollak Y. Ovadia H. Goshen I. Gurevich R. Monsa K. Avitsur R.. (2000). Behavioral aspects of experimental autoimmune encephalomyelitis. J. Neuroimmunol. 104, 31–36. 10.1016/s0165-5728(99)00257-x 10683512

[B118] Pollak Y. Ovadia H. Orion E. Weidenfeld J. Yirmiya R. (2003). The EAE-associated behavioral syndrome: I. Temporal correlation with inflammatory mediators. J. Neuroimmunol. 137, 94–99. 10.1016/s0165-5728(03)00075-4 12667652

[B119] Rademacher D. J. Hillard C. J. (2007). Interactions between endocannabinoids and stress-induced decreased sensitivity to natural reward. Prog. Neuropsychopharmacol. Biol. Psychiatry 31, 633–641. 10.1016/j.pnpbp.2006.12.013 17258369 PMC1876712

[B120] Rademacher D. J. Meier S. E. Shi L. Ho W. S. Jarrahian A. Hillard C. J. (2008). Effects of acute and repeated restraint stress on endocannabinoid content in the amygdala, ventral striatum, and medial prefrontal cortex in mice. Neuropharmacology 54, 108–116. 10.1016/j.neuropharm.2007.06.012 17675104

[B121] Raison C. L. Capuron L. Miller A. H. (2006). Cytokines sing the blues: inflammation and the pathogenesis of depression. Trends Immunol. 27, 24–31. 10.1016/j.it.2005.11.006 16316783 PMC3392963

[B122] Reichenberg A. Yirmiya R. Schuld A. Kraus T. Haack M. Morag A.. (2001). Cytokine-associated emotional and cognitive disturbances in humans. Arch. Gen. Psychiatry 58, 445–452. 10.1001/archpsyc.58.5.445 11343523

[B123] Riazi K. Galic M. A. Kuzmiski J. B. Ho W. Sharkey K. A. Pittman Q. J. (2008). Microglial activation and TNFalpha production mediate altered CNS excitability following peripheral inflammation. Proc. Natl. Acad. Sci. U.S.A. 105, 17151–17156. 10.1073/pnas.0806682105 18955701 PMC2579393

[B124] Rigotti N. A. Gonzales D. Dale L. C. Lawrence D. Chang Y. for the C. I. R. R. U. S. Study Group (2009). A randomized controlled trial of adding the nicotine patch to rimonabant for smoking cessation: efficacy, safety and weight gain. Addiction 104, 266–276. 10.1111/j.1360-0443.2008.02454.x 19149823

[B125] Rossi S. De Chiara V. Musella A. Kusayanagi H. Mataluni G. Bernardi G.. (2008). Chronic psychoemotional stress impairs cannabinoid-receptor-mediated control of GABA transmission in the striatum. J. Neurosci. 28, 7284–7292. 10.1523/JNEUROSCI.5346-07.2008 18632932 PMC6670398

[B126] Rossi S. Furlan R. De Chiara V. Muzio L. Musella A. Motta C.. (2011). Cannabinoid CB1 receptors regulate neuronal TNF-α effects in experimental autoimmune encephalomyelitis. Brain Behav. Immun. 25, 1242–1248. 10.1016/j.bbi.2011.03.017 21473912

[B127] Rossi S. Sacchetti L. Napolitano F. De Chiara V. Motta C. Studer V.. (2012). Interleukin-1β causes anxiety by interacting with the endocannabinoid system. J. Neurosci. 32, 13896–13905. 10.1523/JNEUROSCI.1515-12.2012 23035099 PMC6704788

[B128] Schiffer R. B. Babigian H. M. (1984). Behavioral disorders in multiple sclerosis, temporal lobe epilepsy and amyotrophic lateral sclerosis. An epidemiologic study. Arch. Neurol. 41, 1067–1069. 10.1001/archneur.1984.04050210065016 6477214

[B129] Schubert D. S. Foliart R. H. (1993). Increased depression in multiple sclerosis: a meta-analysis. Psychosomatics 34, 124–130. 10.1016/s0033-3182(93)71902-7 8456154

[B130] Sidhpura N. Parsons L. H. (2011). Endocannabinoid-mediated synaptic plasticity and addiction-related behavior. Neuropharmacology 61, 1070–1087. 10.1016/j.neuropharm.2011.05.034 21669214 PMC3176941

[B131] Silvestri C. Di Marzo V. (2013). The endocannabinoid system in energy homeostasis and the etiopathology of metabolic disorders. Cell Metab. 17, 475–490. 10.1016/j.cmet.2013.03.001 23562074

[B132] Simiand J. Keane M. Keane P. E. Soubrié P. (1998). SR 141716, a CB1 cannabinoid receptor antagonist, selectively reduces sweet food intake in marmoset. Behav. Pharmacol. 9, 179–181. 10065938

[B133] Stella N. (2009). Endocannabinoid signaling in microglial cells. Neuropharmacology 56, 244–253. 10.1016/j.neuropharm.2008.07.037 18722389 PMC2654419

[B134] Strawbridge R. Arnone D. Danese A. Papadopoulos A. Herane Vives A. Cleare A. J. (2015). Inflammation and clinical response to treatment in depression: a meta-analysis. Eur. Neuropsychopharmacol. 25, 1532–1543. 10.1016/j.euroneuro.2015.06.007 26169573

[B135] Su F. Yi H. Xu L. Zhang Z. (2015). Fluoxetine and S-citalopram inhibit M1 activation and promote M2 activation of microglia *in vitro*. Neuroscience 294, 60–68. 10.1016/j.neuroscience.2015.02.028 25711936

[B136] Suh Y. Motl R. W. Mohr D. C. (2010). Physical activity, disability, and mood in the early stage of multiple sclerosis. Disabil. Health J. 3, 93–98. 10.1016/j.dhjo.2009.09.002 21122774

[B137] Thielscher C. Thielscher S. Kostev K. (2013). The risk of developing depression when suffering from neurological diseases. Ger. Med. Sci. 11, 1–7. 10.3205/000170 23326249 PMC3546419

[B138] Tóth A. Blumberg P. M. Boczán J. (2009). Anandamide and the vanilloid receptor (TRPV1). Vitam Horm. 81, 389–419. 10.1016/S0083-6729(09)81015-7 19647120

[B139] Traynor K. (2007). Panel advises against rimonabant approval. Am. J. Health Syst. Pharm. 64, 1460–1461. 10.2146/news070065 17617490

[B140] Trezza V. Baarendse P. J. J. Vanderschuren L. J. M. J. (2010). The pleasures of play: pharmacological insights into social reward mechanisms. Trends Pharmacol. Sci. 31, 463–469. 10.1016/j.tips.2010.06.008 20684996 PMC2946511

[B141] Turna J. Patterson B. Van Ameringen M. (2017). Is cannabis treatment for anxiety, mood, and related disorders ready for prime time? Depress. Anxiety 34, 1006–1017. 10.1002/da.22664 28636769

[B142] Tyndale R. F. Payne J. I. Gerber A. L. Sipe J. C. (2007). The fatty acid amide hydrolase C385A (P129T) missense variant in cannabis users: studies of drug use and dependence in Caucasians. Am. J. Med. Genet. B Neuropsychiatr. Genet. 144B, 660–666. 10.1002/ajmg.b.30491 17290447

[B143] Tyring S. Gottlieb A. Papp K. Gordon K. Leonardi C. Wang A.. (2006). Etanercept and clinical outcomes, fatigue, and depression in psoriasis: double-blind placebo-controlled randomized phase III trial. Lancet 367, 29–35. 10.1016/S0140-6736(05)67763-X 16399150

[B144] Urigüen L. Pérez-Rial S. Ledent C. Palomo T. Manzanares J. (2004). Impaired action of anxiolytic drugs in mice deficient in cannabinoid CB1 receptors. Neuropharmacology 46, 966–973. 10.1016/j.neuropharm.2004.01.003 15081793

[B145] Uriguen L. Perez-Rial S. Ortiz S. Oliva J. M. Palomo T. Manzanares J. (2003). Altered emotional states and impaired anxiolytic action of benzodiacepines in mice lacking cannabinoid CB1 receptors. Eur. Neuropsychopharm. 13, S373 10.1016/S0924-977X(03)92166-3

[B146] van Dam A. M. Brouns M. Louisse S. Berkenbosch F. (1992). Appearance of interleukin-1 in macrophages and in ramified microglia in the brain of endotoxin-treated rats: a pathway for the induction of nonspecific symptoms of sickness? Brain Res. 588, 291–296. 10.1016/0006-8993(92)91588-6 1393581

[B147] Van Gaal L. F. Rissanen A. M. Scheen A. J. Ziegler O. Rössner S. for the RIO-Europe Study Group. (2005). Effects of the cannabinoid-1 receptor blocker rimonabant on weight reduction and cardiovascular risk factors in overweight patients: 1-year experience from the RIO-Europe study. Lancet 365, 1389–1397. 10.1016/S0140-6736(05)66374-X 15836887

[B148] Volkow N. D. Baler R. D. Compton W. M. Weiss S. R. B. (2014). Adverse health effects of marijuana use. N. Engl. J. Med. 370, 2219–2227. 10.1056/NEJMra1402309 24897085 PMC4827335

[B149] Wager T. D. Scott D. J. Zubieta J. K. (2007). Placebo effects on human mu-opioid activity during pain. Proc. Natl. Acad. Sci. U.S.A. 104, 11056–11061. 10.1073/pnas.0702413104 17578917 PMC1894566

[B150] Walsh Z. Gonzalez R. Crosby K. Thiessen M. S. Carroll C. Bonn-Miller M. O. (2017). Medical cannabis and mental health: a guided systematic review. Clin. Psychol. Rev. 51, 15–29. 10.1016/j.cpr.2016.10.002 27816801

[B151] Wang X. Dow-Edwards D. Keller E. Hurd Y. L. (2003). Preferential limbic expression of the cannabinoid receptor mRNA in the human fetal brain. Neuroscience 118, 681–694. 10.1016/S0306-4522(03)00020-4 12710976

[B152] Ward S. J. Dykstra L. A. (2005). The role of CB1 receptors in sweet versus fat reinforcement: effect of CB1 receptor deletion, CB1 receptor antagonism (SR141716A) and CB1 receptor agonism (CP-55940). Behav. Pharmacol. 16, 381–388. 10.1097/00008877-200509000-00010 16148442

[B153] Whiting P. F. Wolff R. F. Deshpande S. Di Nisio M. Duffy S. Hernandez A. V.. (2015). Cannabinoids for medical use: a systematic review and meta-analysis. JAMA 313, 2456–2473. 10.1001/jama.2015.6358 26103030

[B154] Wilson R. I. Nicoll R. A. (2002). Endocannabinoid signaling in the brain. Science 296, 678–682. 10.1126/science.1063545 11976437

[B155] Zeher H. Amin M. E. Rakhawy M. Y. (2010). Coping with depression and anxiety in patients with psoriasis. Egypt. J. Psychiatry 31, 57–63.

[B156] Zhang H. T. Huang Y. Masood A. Stolinski L. R. Li Y. Zhang L.. (2008). Anxiogenic-like behavioral phenotype of mice deficient in phosphodiesterase 4B PDE4B. Neuropsychopharmacology 33, 1611–1623. 10.1038/sj.npp.1301537 17700644 PMC2728355

[B157] Zhang L. Xiong W. (2009). Modulation of the Cys-loop ligand-gated ion channels by fatty acid and cannabinoids. Vitam. Horm. 81, 315–335. 10.1016/S0083-6729(09)81012-1 19647117

[B158] Zhang P. W. Ishiguro H. Ohtsuki T. Hess J. Carillo F. Walther D.. (2004). Human cannabinoid receptor 1: 5′ exons, candidate regulatory regions, polymorphisms, haplotypes and association with polysubstance abuse. Mol. Psychiatry 9, 916–931. 10.1038/sj.mp.4001560 15289816

[B159] Zorrilla E. P. Luborsky L. McKay J. R. Rosenthal R. Houldin A. Tax A.. (2001). The relationship of depression and stressors to immunological assays: a meta-analytic review. Brain Behav. Immun. 15, 199–226. 10.1006/brbi.2000.0597 11566046

[B160] Zuo L. Kranzler H. R. Luo X. Covault J. Gelernter J. (2007). CNR1 variation modulates risk for drug and alcohol dependence. Biol. Psychiatry 62, 616–626. 10.1016/j.biopsych.2006.12.004 17509535

